# Subjective memory concern, negative affect, and cortical microstructural integrity in community-dwelling middle-aged men

**DOI:** 10.1007/s11357-025-01778-4

**Published:** 2025-07-08

**Authors:** Rongxiang Tang, Tyler R. Bell, Jeremy A. Elman, Chandra A. Reynolds, Daniel E. Gustavson, Olivia K. Puckett, Matthew S. Panizzon, Rosemary Toomey, Ruth McKenzie, Michael J. Lyons, Donald J. Hagler, Christine Fennema-Notestine, Lisa T. Eyler, Anders M. Dale, Carol E. Franz, William S. Kremen

**Affiliations:** 1 Department of Psychological and Brain Sciences, Texas A&M University, College Station, TX 77843 USA; 2 Department of Psychiatry, University of California San Diego, La Jolla, CA 92093 USA; 3 Present Address: Center for Behavior Genetics of Aging, University of California San Diego, La Jolla, CA 92093 USA; 4 Institute for Behavioral Genetics and Department of Psychology and Neuroscience, University of Colorado Boulder, Boulder, CO 80303 USA; 5 Department of Psychological and Brain Sciences, Boston University, Boston, MA 02215 USA; 6 School of Education and Social Policy, Merrimack College, North Andover, MA 01845 USA; 7 Department of Radiology, University of California San Diego, La Jolla, CA 92093 USA; 8 Department of Neurosciences, University of California San Diego, La Jolla, CA 92093 USA

**Keywords:** Subjective memory concern, Negative affect, Cortical mean diffusivity, Trait anxiety, Depression

## Abstract

Concerns about memory often increase with age and have been suggested as a precursor to impending memory impairment or dementia. However, subjective memory concern (SMC) has also been shown to reflect an individual’s trait-like tendency to worry about memory, which is more strongly linked to negative affect than to objective memory performance. Despite behavioral evidence supporting a trait-like dimension of SMC, its neuroanatomical underpinnings remain underexplored. In 477 community-dwelling dementia-free men (56–72 years old), we investigated the association between SMC and cortical mean diffusivity (cMD)—a diffusion MRI-based metric of gray matter microstructural integrity—generating a brain-wide map of their association. Self-report trait anxiety and depressive symptoms were collected, along with objective memory scores based on three neuropsychological tasks for which brain maps of their association with cMD were also generated. Finally, we conducted spatial correlational analyses to compare the spatial patterns of these brain association maps to assess whether there were significant spatial resemblances between each. We found that the gray matter integrity correlates of SMC spatially resembled those of depressive symptoms and trait anxiety but not those of objective memory. The spatial correspondences between gray matter integrity correlates of negative affect measures and SMC were significantly stronger than those between SMC and objective memory. Together, these results suggest a neuroanatomical basis of trait-like SMC, which should be distinguished from state-related SMC that may be a precursor of objective memory deficits in research and clinical settings.

**Supplementary Information:**

The online version contains supplementary material available at 10.1007/s11357-025-01778-4.

## Introduction

Memory concerns are common among older adults, with some reporting persistent self-experienced decline in memory but no decline in objective memory performance as measured by neuropsychological assessments [1]. This self-perceived memory decline is a criterion of subjective cognitive decline (SCD), which is considered to be a precursor to mild cognitive impairment (MCI) and Alzheimer’s disease (AD) [1, 2]. However, some studies have shown that subjective memory concern (SMC) does not correspond well to either concurrent objective memory performance or objective memory decline [3–7]. In fact, emerging evidence has indicated that SMC likely includes both trait-like and state-like dimensions [8], with the former reflecting the inherent predisposition to worry more or less about one’s memory [8–10], and the latter reflecting an elevated risk of impending cognitive decline or AD that is of clinical relevance [1, 2]. Thus, gaining a better understanding of trait-like versus state-like dimensions of SMC is critical for accurately identifying individuals at risk of impending cognitive decline and dementia in clinical and research settings.

The trait-like dimension of SMC has largely been overlooked, as SMC has predominately been examined as a state-related construct. Prior work by our group has shown that not only was there a stable trait-like dimension of SMC that is heritable as early as age 38 and remains heritable three decades later, but this trait-like dimension is also genetically correlated with depressive symptoms and trait anxiety, such that shared genetic influences contribute to variance in SMC and these measures of negative affect [8]. Moreover, SMC demonstrated a stronger genetic correlation with negative affect than with objective memory [8], which is consistent with prior studies that showed stronger phenotypic correlations of SMC with negative affect than with objective memory performance [6, 11–13]. These behavioral findings suggest that SMC can partly reflect negative affect, which contributes to the trait-like component, rather than reflecting actual memory decline [6, 13]. However, less is known about the link between SMC and negative affect within the brain, and whether there are overlapping neuroanatomical correlates between SMC and negative affect that may explain their shared variance.

Many neuroanatomical studies of SCD, which include SMC as one defining characteristic of SCD, have focused on macrostructural measures of gray matter neurodegeneration such as cortical thickness and volume. For instance, reduced cortical thickness of entorhinal cortex and reduced hippocampal volume are observed in those with SCD relative to healthy controls [14]. However, these gray matter macrostructural measures are less sensitive to early AD-related neurodegeneration and pathological processes than gray matter microstructural measures derived from diffusion imaging [15–18]. Specifically, cortical mean diffusivity (cMD), a microstructural measure of water diffusion in gray matter often used to index gray matter microstructural integrity [19], has been shown to exhibit more widespread gray matter changes than cortical thickness in individuals without dementia who are in their early 60 s [15, 16]. Most importantly, the spatial and temporal patterns of cMD changes are more strongly associated with AD pathology than cortical thickness changes [15, 17]. Considering that individuals with SMC are often cognitively unimpaired based on standard neuropsychological tests, cMD may be an earlier and more sensitive neuroanatomical measure than macrostructural measures for examining SMC-related gray matter alterations. Moreover, to our knowledge, no study has examined the association between SMC and gray matter microstructure, suggesting the need for in-depth investigations.

In the present study, we first investigated the neuroanatomical correlates of SMC in 477 community-dwelling dementia-free men aged 56 to 72 years old by examining the associations of current SMC and SMC assessed on average at age 38 with gray matter microstructural integrity of 200 cortical parcels as indexed by cMD. Age 38 SMC was included as our group has previously found it to reflect the trait-like dimension of SMC [8]. Next, we used the same approach to separately examine the neuroanatomical correlates of negative affect (i.e., depressive symptoms and trait anxiety) and objective memory performance. After examining parcel-wise/regional correlates and obtaining the brain maps of associations for SMC (current and age 38), negative affect measures, and objective memory performance, we systematically compared the spatial patterns of these brain association maps to assess whether there were significant spatial resemblances between each. We used spatial correlational analysis, an established and widely used method for comparing spatial correspondence between brain maps at the whole brain level [20]. Specifically, we investigated whether brain regions that show lower gray matter microstructural integrity associated with higher current SMC also tend to show lower gray matter microstructural integrity associated with higher negative affect and lower objective memory. Based on prior behavioral investigations of SMC, we hypothesized that there would be a link between current SMC and negative affect in the brain, such that there would be greater spatial resemblance of brain association maps between current SMC and negative affect measures than between current SMC and objective memory performance. Moreover, there would be greater spatial resemblance of brain association maps between current and age 38 SMC than between current SMC and objective memory performance, considering the behavioral evidence that supports a stable trait-like SMC, which remains heritable from age 38 into late midlife.

## Methods

### Participants

Participants were in the Vietnam Era Twin Study of Aging (VETSA), an ongoing multisite longitudinal study of aging and risk for AD beginning in middle age [21]. Specifically, participants were men in the Vietnam Era Twin Registry, which includes only male-male twin pairs because, at that time, there were too few female twins (1965–1975) to be included in data analysis from the Registry. Participants are similar to American men in their age cohort with respect to health, education, and lifestyle characteristics [22]. VETSA has completed three waves of assessments, but the present study was a cross-sectional analysis. In the present study, the sample included 477 community-dwelling men (mean age = 63.37 years, SD = 3.64; range = 56–72) from waves 2 and 3, most of whom were non-Hispanic White (88.7%), with only a small percentage identifying as other races/ethnicities (Hispanic White, 2.3%; Black American, 7.3%; American Indian/more than one race, 1.7%). Wave 1 participants were not included because the neuroimaging was conducted on 1.5 T rather than 3 T MRI scanners. For each participant, we only included their first 3 T neuroimaging data and the concurrent behavioral, and cognitive assessments. All participants traveled to the University of California, San Diego (UCSD) or Boston University (BU) to participate in the VETSA. Informed consent was obtained from all participants and institutional review boards at both sites approved all protocols. Table 1 shows the sample characteristics.

**Table 1:** Characteristics of the study sample

Characteristics	Mean (SD) *N* = 477		Range	
Age at Assessment	63.37 (3.64)		55.96–71.72	
Years of Education	13.91 (2.06)		8.00–20.00	
Subjective Memory Concern*				
*Age 38*^*a*^	1.80 (0.93)		1.00–5.00	
*Current*^*a*^				
*Rating of Memory Problem*	1.79 (0.61)		1.00–4.00	
*Concern about Memory*	1.98 (0.80)		1.00–4.00	
Objective Episodic Memory^b^	− 0.31 (1.04)		− 4.27–2.52	
Depressive Symptoms*	6.47 (6.74)		0.00–39.00	
Trait Anxiety*	30.64 (9.10)		20.00–65.00	

*Missing: Age 38 Subjective Memory Concern (*N* = 16), Current Subjective Memory Concern (*N* = 2), Depressive Symptoms (*N* = 4), Trait Anxiety (*N* = 3)

^a^Higher scores of subjective memory concern indicate greater concern. Current subjective memory concern is a latent factor score derived from 2 memory items including rating of memory problem and concern about memory

^b^Objective episodic memory is a factor score that was standardized to wave 1 mean and sd. Lower scores indicate worse episodic memory performance

### Age 38 subjective memory concern

SMC was assessed using self-report items. Prior to the VETSA assessments and at a mean age of 38 years, participants completed the Survey of Health study [23], which had a single SMC question (“In the last 6 months have you had trouble with your memory?”). Participants were asked to respond on a 5-point Likert-type scale from “1 – Very often” to “5 – Never.” This item was reverse scored, so that higher ratings indicate more SMC. Hereafter, we referred to this as age 38 SMC.

### Current measures

SMC, VETSA objective memory, depressive symptoms, and trait anxiety measures were assessed at the same visit as the MRI/cMD measure and are described as follows.

### Subjective memory concern

In VETSA, SMC was assessed using two items (“In general, rate your memory in terms of the kinds of problems you have,” “In general, how concerned are you about your memory?”), which were rated on a 4-point Likert-type scale so that higher values indicate more SMC (“1 – No problems/Not at all concerned” to “4 – Major problems/Very Concerned”). Responses were then combined into a factor score as done previously [8]. Hereafter, we referred to this as current SMC. Higher scores represent greater concerns.

### Objective memory

Objective memory was assessed during VETSA using well-established neuropsychological tests: the Wechsler Memory Scale-III [24] Logical Memory and Visual Reproduction subtests (immediate and delayed recall conditions) and the California Verbal Learning Test-II [25] (short- and long-delay free recall conditions and the total number of words recalled across the five learning trials). An episodic memory factor score based on these seven measures was computed for each participant using confirmatory factor analyses [26]. All scores for returning participants were adjusted for practice effects as described previously [27]. Higher scores represent better current objective memory performance.

### Negative affect

#### Depressive symptoms

The Center for Epidemiological Studies-Depression Scale was used for measuring current depressive symptoms, which is a 20-item self-report questionnaire that is widely used in the general population [28].

#### Trait anxiety

Following our previous SMC work [8], the trait form of the State-Trait Anxiety Inventory was used to assess current trait anxiety, which includes 20 items [29].

 For all negative affect measures, higher scores represent higher levels of depressive symptoms and trait anxiety.

### MRI acquisition and processing

T1-weighted and diffusion-weighted images were acquired at UCSD (*N* = 341) and at Massachusetts General Hospital (*N* = 136). At UCSD, T1-weighted (3D fast spoiled gradient echo, TR = 8.084 ms, TE = 3.164 ms, in-plane resolution = 1 × 1 mm, slice thickness = 1.2 mm) and diffusion-weighted images (51 diffusion directions, *b* value = 1000 s/mm^2^, integrated with a pair of *b* = 0 images with opposite phase-encode polarity, TR = 9700 ms, TE = 80–84 ms, in-plane resolution = 2.5 × 2.5 mm, slice thickness = 2.5 mm) were acquired on two 3 T GE Discovery 750 × scanners with an eight-channel phased array head coil. At MGH, T1-weighted (3D magnetization-prepared rapid gradient-echo, TR = 2170 ms, TE = 4.33 ms, in-plane resolution = 1 × 1 mm, slice thickness = 1.2 mm) and diffusion-weighted images (30 diffusion directions, *b* value = 1000 s/mm^2^, one *b* = 0 image, TR = 9500 ms, TE = 94 ms, in-plane resolution = 2.5 × 2.5 mm, slice thickness = 2.5 mm) were acquired with a 3 T Siemens Tim Trio with a 32-channel head coil. In addition, a separate pair of *b* = 0 images with opposite phase-encode polarity, used for B0 distortion correction, were acquired on the Siemens scanner.

All images were preprocessed at the UCSD Center for Multimodal Imaging Genetics and were visually inspected to exclude data with severe scanner artifacts or excessive head motion from subsequent analyses as described previously [30]. Briefly, T1-weighted images were corrected for gradient nonlinearity distortions and B1 field inhomogeneity and then rigidly resampled and registered to standard space with 1 mm isotropic voxel size. Diffusion-weighted images (DWI) were corrected for eddy current distortion, head motion, B0 distortions, and gradient nonlinearity distortions. The *b* = 0 images were registered to T1 images using mutual information and then rigidly resampled into a standard orientation relative to the atlas-registered T1, with 2 mm isotropic resolution. Boundaries between gray matter, white matter, and cerebrospinal fluid were defined using Freesurfer 6.0 [31] and the cortical surface was divided into 200 different parcels (100 per hemisphere) according to the Schaefer 200 atlas [32]. Conventional diffusion tensor imaging (DTI) methods were used to model the diffusion tensor as an ellipsoid. Eigenvalues λ1, λ2, and λ3 quantify the diffusivity of the three primary axes, respectively, and mean diffusivity (MD) was calculated as the average of the three eigenvalues in each voxel. To minimize the effects of partial voluming, we projected values of MD to the cortical surface by taking the weighted average of multiple samples through the cortical ribbon at each vertex, with weights based on the proportion of gray matter in each voxel sampled as described previously [30]. Cortical MD was then calculated as the average across vertices for each cortical parcel.

### Statistical analysis

**Fig. 1:**
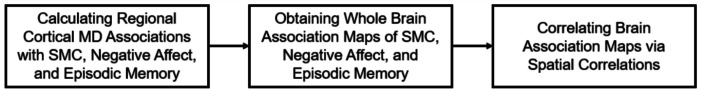
An overview of analytic steps

Statistical analyses were performed using R version 4.1.2, with the exception of spatial correlations and permutation tests, which were performed using the BrainStat toolbox (Larivière et al., 2023) (https://github.com/MICA-MNI/BrainStat). Linear mixed models were performed using the lme4 package [33], and *p* values were calculated using Satterthwaite degrees of freedom approximation. Multiple comparison correction was applied using the Benjamini–Hochberg false discovery rate (FDR) control. Outliers were winsorized to the nearest minimum or maximum data point if the data points were 3 standard deviations or more above or below the mean. An overview of the analyses is depicted in Fig. 1.

### Subjective memory concern, negative affect, and objective memory associations with cortical mean diffusivity

To examine the associations of SMC and negative affect measures with cMD, we built a linear mixed model for each of the 200 cortical parcels with each of the following variables as the predictor of interest in separate models: SMC (age 38 or current), depressive symptoms, and trait anxiety. cMD of each parcel was the dependent variable. Age and scanner were included as covariates in all models. Twin pair family ID was included as a random intercept in all models to account for correlated outcomes within pairs. Specifically, the linear mixed models are as follows: cMD_parcel_ ~ β_0_ + β_2_*Predictor + β_2_*Age + β_3_*Scanner + (1|Twin Pair ID) where cMD_parcel_ is cortical mean diffusivity within each parcel, Predictor is our predictor of interest (i.e., Age 38 SMC, current SMC, depressive symptoms, or trait anxiety), and twin pair ID is used as a random intercept to account for correlated outcomes between twins. The resulting statistical maps of β2 corresponding to the regional associations between the predictor of interest and cortical MD were used to test the spatial correspondence of effects. The standardized beta coefficient of the predictor for each parcel was extracted to index the magnitude of association between predictor and cMD. We then projected these standardized beta coefficient values onto the cortical surface, yielding a group-level brain map for each of the five predictors of interest. FDR-corrected *p* values for the standardized beta coefficients of each predictor were extracted from all 200 parcels to determine which parcels were significantly associated with the predictors of interest.

### Spatial correlations and permutation tests

Spatial correlations among group-level brain maps were determined by calculating the correlation of values (i.e., standardized beta coefficients) across all parcels between two given brain maps. Because the intrinsic spatial smoothness in brain maps may inflate the significance of their spatial correlation, the statistical significance of these correlation coefficients was assessed via spatial autocorrelation-preserving permutation tests or “spin tests” [20].

## Results

### Correlations among variables of interest

Consistent with our prior observations in the full sample of VETSA participants including those without MRI data [8], older age was significantly correlated with lower levels of current objective memory performance (*r* = − 0.15, *p* < 0.01) and nearly so with levels of current SMC (*r* = − 0.09, *p* = 0.06). However, higher levels of current SMC were associated with lower levels of current objective memory performance (*r* = − 0.11, *p* = 0.01). Age 38 SMC was positively correlated with current SMC (*r* = 0.21, *p* < 0.01), but not with current objective memory performance (*r* = − 0.04, *p* = 0.36). For negative affect measures, higher levels of current trait anxiety and depressive symptoms were associated with higher levels of current SMC (*r* = 0.40, 0.37, respectively, *p*s < 0.01), higher levels of age 38 SMC (*r* = 0.32, 0.29, respectively, *p*s < 0.01), and with lower levels of current objective memory (*r* = − 0.17, − 0.13, respectively, *p*s < 0.01).

### Associations between subjective memory concern and cortical microstructural integrity

**Fig. 2:**
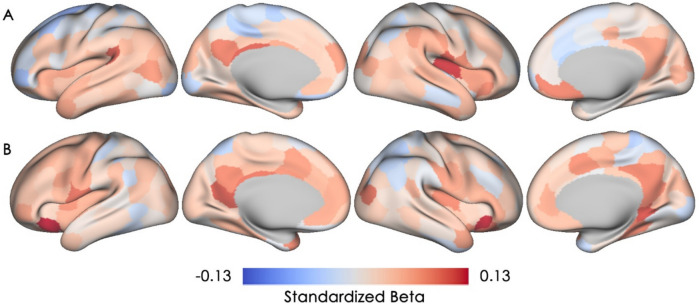
Associations of age 38 and current subjective memory concern with cortical mean diffusivity. Standardized beta coefficients are plotted for all 200 cortical parcels, with warmer color indicating a positive association (i.e., lower microstructural integrity) and cooler color indicating a negative association (i.e., higher microstructural integrity). None of these parcels remained significant after FDR-correction.** A** Association between age 38 subjective memory concern and cortical mean diffusivity is depicted. **B** Association between current subjective memory concern and cortical mean diffusivity is depicted

The magnitudes and directions of the associations of age 38 and current SMC with current cMD are shown in Fig. 2. Across the neocortex, higher levels of age 38 SMC were associated with higher cMD (i.e., lower cortical microstructural integrity) in the left parietal operculum, right somatosensory network regions, and left posterior cingulate cortex/Brodmann area (BA) 23 (four parcels with standardized beta ≥ 0.1, Fig. 2A). Higher current SMC was associated with higher cMD, particularly in the bilateral ventral prefrontal cortex/BA 47, the left precuneus/posterior cingulate cortex, and visual cortex/BA 19 (four parcels with a small effect size of standardized beta ≥ 0.1, Fig. 2B). However, none of these parcels remained statistically significant after FDR correction (uncorrected p value maps can be found in the Supplementary Materials).

### Association between current objective memory and cortical microstructural integrity

**Fig. 3:**
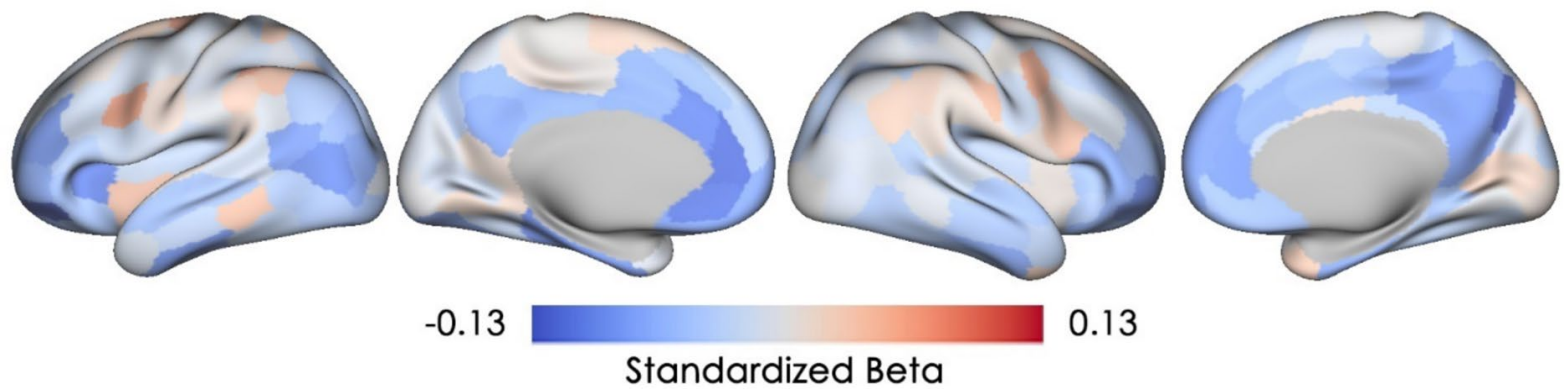
Associations of objective memory with cortical mean diffusivity. Standardized beta coefficients are plotted for all 200 cortical parcels, with warmer color indicating a positive association (i.e., lower microstructural integrity) and cooler color indicating a negative association (i.e., higher microstructural integrity). None of these parcels remained significant after FDR-correction

Lower current objective memory performance was associated with higher cMD (i.e., lower microstructural integrity) in regions within the left orbitofrontal cortex, left anterior cingulate cortex/BA 23/32, and right precuneus (four parcels with a small effect size of standardized beta ≥ absolute value [0.1], Fig. 3). However, none of these parcels remained statistically significant after FDR correction and uncorrected *p* value maps can be found in the Supplementary Materials.

### Associations between negative affect and cortical microstructural integrity

**Fig. 4:**
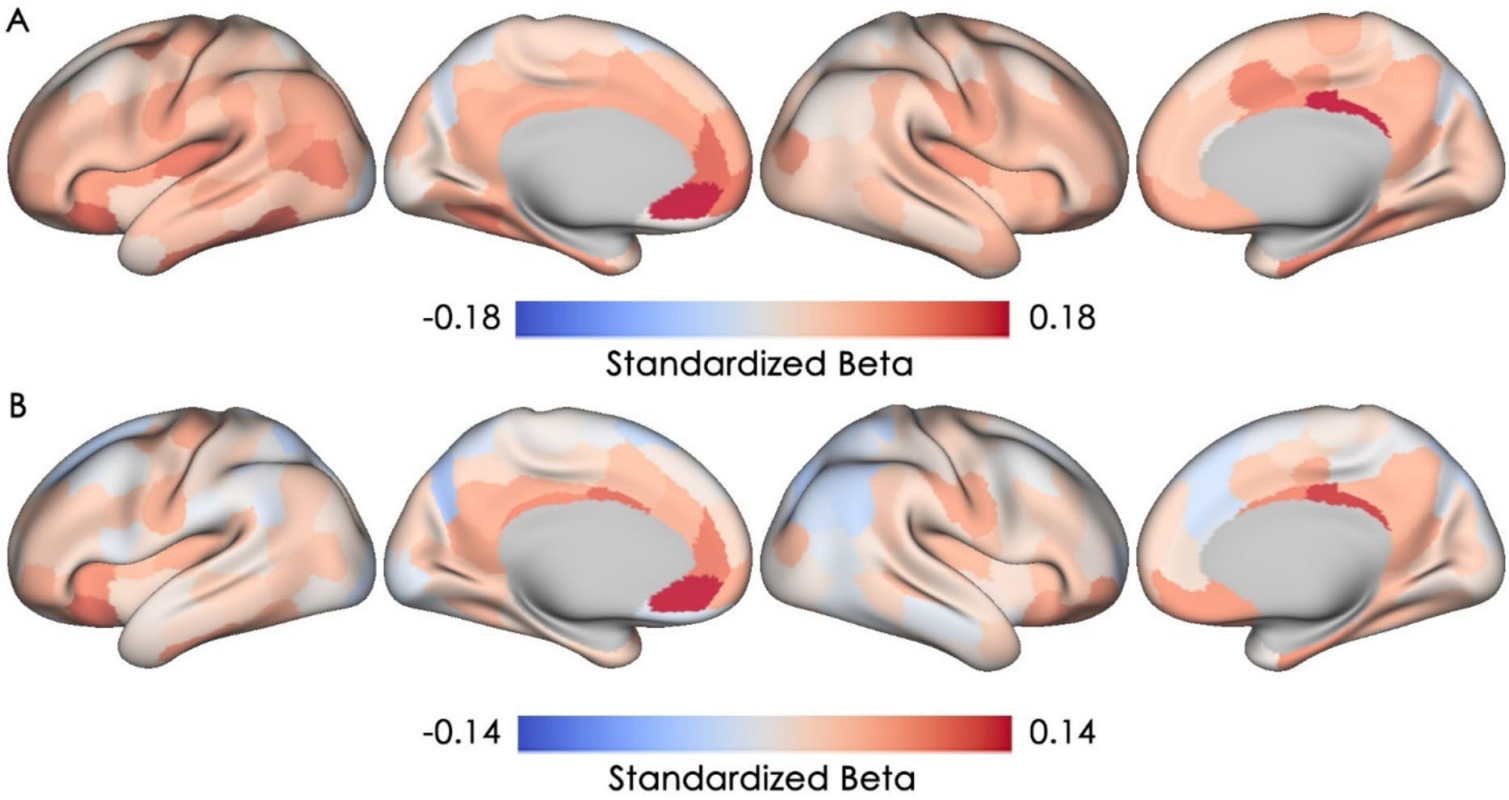
Associations of negative affect with cortical mean diffusivity. Standardized beta coefficients are plotted for all 200 cortical parcels, with warmer color indicating a positive association (i.e., lower microstructural integrity) and cooler color indicating a negative association (i.e., higher microstructural integrity).** A** Association between depressive symptoms and cortical mean diffusivity is depicted. **B** Association between trait anxiety and cortical mean diffusivity is depicted

The magnitudes and directions of the associations between the two measures of negative affect and cMD are shown in Fig. 4. Higher depressive symptoms were associated with higher cMD across the neocortex, with 27 regions in the bilateral posterior cingulate cortex, temporal pole, left insular cortex, right orbitofrontal cortex, left anterior cingulate cortex, and left ventromedial prefrontal cortex showing at least small effect sizes (standardized beta ≥ 0.1) and 17 of the 27 regions showing significant associations after FDR correction (see Supplementary Materials for a list and a figure of significant parcels). Notably, most of these parcels belong to the default mode, limbic, and frontoparietal control networks. Similarly, higher levels of trait anxiety were associated with higher cMD in the left ventral prefrontal cortex/Brodmann 47, left ventromedial prefrontal cortex, left mid-cingulate cortex, and right posterior cingulate cortex (4 parcels with standardized beta ≥ 0.1). However, none of these parcels remained statistically significant after FDR correction. Overall, for both depressive symptoms and trait anxiety, the left ventromedial prefrontal cortex, left ventral prefrontal cortex/BA 47, and right posterior cingulate cortex showed consistent associations such that higher levels of negative affect were related to lower cortical microstructural integrity in all of these regions.

### Spatial correspondence between brain association maps of subjective memory concern and negative affect measures with cortical microstructural integrity

To investigate whether regions that exhibited lower cortical microstructural integrity associated with higher levels of current SMC also tended to exhibit lower integrity associated with higher levels of negative affect measures, we conducted spatial correlational analyses to compare the spatial similarity between these brain maps (i.e., spatial patterns of associations). Specifically, we found that the spatial patterns of associations of current SMC and trait anxiety with cMD were indeed positively correlated (*r* = 0.56, *P*_FDR-corrected_ = 0.0002), such that regions that exhibited lower cortical microstructural integrity associated with higher SMC also tended to be associated with higher levels of trait anxiety. Likewise, the spatial patterns of associations of current SMC and depressive symptoms associated with cMD were significantly correlated (*r* = 0.45, *P*_FDR-corrected_ = 0.0002).

However, we did not detect a significant spatial correspondence between association maps of current SMC and objective memory performance (*r* = − 0.11, *P*_FDR-corrected_ = 0.2267). Moreover, the spatial correlations of current SMC with trait anxiety and depressive symptoms were significantly stronger than the correlation of current SMC with objective memory performance (trait anxiety, 0.56 vs. − 0.11[absolute value]: *z* = 8.02, *p* < 0.0001, 2-tailed; depressive symptomsm 0.45 vs. − 0.11[absolute value]: *z* = 5.74, *p* < 0.0001, 2-tailed).

Interestingly, the association maps of current SMC and age 38 SMC were positively correlated (*r* = 0.33, *P*_FDR-corrected_ = 0.0006) such that regions that exhibited lower microstructural integrity associated with higher levels of current SMC tended to be the ones that exhibited lower microstructural integrity associated with higher levels of age 38 SMC. Finally, the spatial correlation of association maps of age 38 SMC with current SMC was also significantly stronger than the spatial correlation of objective memory performance with current SMC (0.33 vs. − 0.11[absolute value]: *z* = 3.54, *p* < 0.05, 2-tailed).

## Discussion

SMC has been associated with negative affect, which partly reflects a trait-like tendency to worry more or less about one’s memory in addition to being a possible indicator of one’s risk of impending cognitive decline and dementia [8]. However, very little attention has been paid to the existence of the trait-like component of subjective memory concern. We demonstrated that across the neocortex, the neuroanatomical correlates of current SMC spatially resembled those of concurrent negative affect including depressive symptoms and trait anxiety but not those of objective memory performance. Moreover, we found that the spatial correspondences between neuroanatomical correlates of current SMC and negative affect, and current and age 38 SMC, were significantly stronger than the spatial correspondence between current SMC and objective memory performance. Together, these results suggest that there is shared variance between concurrent SMC and negative affect that is observable at the neuroanatomical level across the neocortex. Moreover, these associations are observed even for SMC assessed at an average of 25 years earlier, strongly supporting the existence of a trait-like dimension of SMC with a neuroanatomical basis that needs to be distinguished from state-related SMC in research and clinical settings.

Examining the spatial patterns of brain-behavior associations, we found significant spatial overlap between neuroanatomical correlates of SMC and negative affect. Specifically, trait anxiety showed the strongest spatial similarity with current SMC, followed by the spatial similarity between depressive symptoms and current SMC, both at a magnitude that was significantly stronger than the spatial similarity between current SMC and objective memory performance, which was not significant. Trait anxiety reflects a habitual tendency to worry and feel anxious [34], and has been shown to be influenced partly by the same set of genes that also influence SMC [8]. Thus, the spatial overlap in neuroanatomical correlates between SMC and trait anxiety could be due to the same shared genetic and/or developmental underpinnings, further suggesting the existence of a trait-like dimension of SMC that is linked to negative affect. The spatial similarity between neuroanatomical correlates of depressive symptoms and SMC and the lack of spatial correspondence between objective memory performance and SMC further corroborated the notion that SMC may partly reflect negative affect and not actual current or impending memory decline in our community-dwelling dementia-free cohort of male participants. Moreover, the significant spatial correspondence between neuroanatomical correlates of age 38 and current SMC suggests that there is a trait-like dimension of SMC that is represented in the brain, which remains fairly stable even nearly three decades later. However, it should be noted that age 38 and current SMC were assessed using different items that asked about severity and frequency of memory concerns respectively. This may potentially contribute to measurement inconsistency that may lead to differences in their underlying neuroanatomical correlates, which warrants future longitudinal investigations of SMC. Nonetheless, our prior analyses in this cohort have shown that SMC at age 38 and in later life are significantly and genetically correlated [8], suggesting they, albeit phrased somewhat differently, do still tap into a stable underlying trait-like dimension of SMC. Moreover, although the age 38 item “In the last 6 months have you had trouble with your memory?”) is couched in terms of frequency, it is likely also related to severity given the extensive time period. For example, it seems likely that a response of “Very often” would also indicate greater severity. Relatedly, it should be noted that subjective cognitive concerns have been shown to be more prevalent and more strongly associated with depressive symptoms in women than in men [35, 36]. Although there hasn’t been any study directly examining sex differences in the association between trait anxiety and SMC, trait anxiety is generally known to be higher in females than in males. Thus, there may be sex differences in the spatial correspondence between neuroanatomical correlates of SMC and negative affect, and in the neuroanatomical correlates themselves, which we could not examine in our sample. Speculatively, the observed spatial correspondence between neuroanatomical correlates of SMC and negative affect may be stronger in women, as the SMC measure likely captures the more trait-like dimension.

Finally, it should be noted that these spatial correlations among brain association maps of behavioral measures should not be interpreted as mere reflections of the behavioral correlations among these measures. The variance shared between these measures at the behavioral level does not necessarily translate to the variance shared between the neural representations or brain regions associated with these behavioral measures. In other words, two behavioral measures could be correlated (e.g., memory and executive function) but the spatial patterns of their neuroanatomical underpinnings could be very different. Thus, the fact that we observed spatial correspondence between brain regions associated with SMC and negative affect measures suggests that the behavioral measures are correlated at least partially because they have similar neuroanatomical underpinnings. Collectively, our neuroanatomical results strengthen prior behavioral findings that the trait-like component of SMC is linked to negative affect with shared genetic underpinnings that should be separated from its state-like dimension.

Although parcel-level associations are not the focus of our study, we found that higher current SMC was linked to lower cortical microstructural integrity in bilateral BA 47 implicated in negative emotion processing and recognition [37] and the right precuneus/PCC implicated in episodic memory [38], with at least small effect sizes. The integrity of left BA 47 was also negatively associated with depressive symptoms and trait anxiety in our community-dwelling, non-clinical sample, which is in line with work showing reduced cortical thickness in BA 47 in major depressive disorder patients relative to healthy controls [39]. More broadly, BA47 is also actively involved in emotion regulation processes, with increased functional activation during the reappraisal of negative emotions or the reduction of negative affect as demonstrated by functional MRI studies [40]. Speculatively, the reduced microstructural integrity in BA47 may impair emotion regulation, potentially contributing to greater negative affect. However, the right precuneus/PCC was not associated with negative affect measures. The precuneus/PCC is a site of early accumulation of AD pathology [41] and structural deterioration in precuneus/PCC has been well-documented in individuals with cognitive impairment and dementia [17, 42]. A previous study in MCI patients showed that those with depressive symptoms had relatively preserved brain volume in the isthmus of the cingulate gyrus adjacent to the PCC compared to MCI patients without depressive symptoms [43], further reinforcing the notion that the trait-like SMC is linked to negative affect and likely does not affect regions implicated in AD. Thus, the lack of association between right precuneus/PCC and negative affect is not surprising and may suggest that its cortical microstructural integrity reflects potential variations in the state-like dimension of SMC and reduced integrity may presage future memory decline.

Finally, although we did not detect significant regional associations between current SMC and cortical microstructural integrity, the small effect sizes are expected and are consistent with prior findings in the neuroimaging literature. For instance, a previous large-scale neuroanatomical study (*N*≈40,000) found significant but also small effect sizes of brain structure and behavior associations [44]. Moreover, a recent analysis showed that small effect sizes are common in neuroimaging studies of brain-behavior associations, particularly in non-clinical populations and even in well-powered studies [45]. With our sample of 477 we had 80% power to detect effect sizes of > 0.13 at α = 0.05 (2-tailed). Although the magnitude of the effects we observed were comparable to those reported in the broader literature, we may thus have been underpowered for them to reach statistical significance after FDR correction.

There were also some general trends in the parcel-level neuroanatomical correlates of negative affect, age 38 SMC, and objective memory performance. Higher depressive symptoms and trait anxiety were associated with lower cortical microstructural integrity of parcels within the default mode and frontoparietal control networks implicated in the processing and recognition of negative affect. Higher age 38 SMC was associated with lower integrity of the left posterior cingulate/BA 23, which plays roles in both emotion and episodic memory processes [46]. Finally, lower objective memory performance was associated with lower integrity of parcels within the left orbitofrontal cortex and right precuneus, which are implicated in episodic memory, temporal context memory and autographical memory with emotional significance [47–49]. Overall, the majority of parcels have functional roles consistent with the behavioral variables with which they were associated.

There are a few limitations to this study. First, our cohort included mostly white and non-Hispanic community-dwelling men, which means results may not be generalizable to women, other racial/ethnic groups, or clinical populations, who may exhibit differences in terms of levels of SMC and negative affect. Nonetheless, our findings provide the initial neuroanatomical evidence supporting a link between SMC and negative affect in men, highlighting the need for future investigations in women, as well as studies that examine both sexes. Specifically, future work would benefit from examining sex as a covariate as well as interaction effects between sex and SMC, or sex and negative affect measures, on cortical microstructural integrity to determine whether sex differences exist. Second, questions about SMC at the two time points were somewhat different, as the former included one question about frequency and the latter included two questions about severity. Despite these differences, we still found good correspondence between age 38 and current SMC assessments. The present findings suggest the need for large-scale longitudinal investigations that begin tracking SMC earlier in life to better separate the trait-like and state-like dimension later in life. Third, we did not examine other brain macrostructural measures (e.g., thickness, volume) that may also be associated with SMC, negative affect, and objective memory, as our focus was on gray matter microstructure given that it is earlier and more sensitive to AD-related processes than gray macrostructural measures. Future studies on SMC and these structural measures are needed.

Taken together, in older adults, subjective reports of memory concern may originate from some mixture of both trait-like tendencies to be concerned about one’s memory that are exacerbated by negative affect, and state-related experiences of actual yet subtle memory difficulty that reflect elevated risk of developing cognitive impairment and dementia. In part, these subtle subjective changes might not initially be associated with objective performance decline, but rather with the experience of requiring more effort to achieve the same level of performance [50]. Our findings of the spatial resemblance between neuroanatomical correlates of SMC and negative affect likely suggests the existence of putative neural bases for the trait-like dimension of SMC, which may inform future neuroimaging studies on differentiating between the trait-like and state-like dimensions of SMC. Additionally, our findings highlight the potential clinical utility of assessing the spatial patterns of neuroanatomical changes in individuals with SMC and comparing those patterns with established patterns of AD-related neurodegeneration and pathology. Our work on cMD changes demonstrated that individuals with a more AD-like pattern of cMD change experienced greater memory decline over 5 years compared to those with a less AD-like pattern [15]. Similarly, longitudinal studies of individuals with SMC might aid clinicians in differentiating neuroanatomical patterns linked to SMC that is associated with AD risk from those that reflect SMC, which is likely associated with negative affect and to be more longstanding. However, prior to examination of neuroanatomical patterns, development of an SMC questionnaire analogous to Speilberger’s state-trait anxiety inventory [29] might turn out to be quite useful to clinicians. More broadly, our findings may suggest that assessing and treating negative affect earlier in life as part of routine primary care could not only facilitate better monitoring and evaluation of the underlying contributions (i.e., trait vs. state) to SMC in clinical settings but also encourage individuals to reduce worrying about their cognitive performance and engage in behaviors that could help them maintain cognitive performance in later life.

## Supplementary Information

Below is the link to the electronic supplementary material.

ESM 1 (DOCX 529 KB)
