# Supplementary material for: Subjective memory concern, negative affect, and cortical microstructural integrity in community-dwelling middle-aged men
Source: GeroScience. 2025 Jul 8;48(2):2267–80. doi: 10.1007/s11357-025-01778-4 (PMC12972471; doi:10.1007/s11357-025-01778-4)

**Supplementary Materials**

## **Table S1.** List of brain parcels with significant association between their cortical mean diffusivity and depressive symptoms. Center of mass and corresponding Schaefer 200, 7 networks label for each parcel is shown.

| Parcel Number | Hemisphere | Network | ROI Name | R | A | S | Corrected P | Uncorrected P |
| --- | --- | --- | --- | --- | --- | --- | --- | --- |
| 1 | Left | Visual | LH_Vis_1 | -24 | -53 | -9 | 0.024957 | 0.001248 |
| 8 | Left | Visual | LH_Vis_8 | -47 | -70 | 10 | 0.034315 | 0.00223 |
| 16 | Left | Somatomotor | LH_SomMot_2 | -53 | -24 | 9 | 0.034315 | 0.002115 |
| 17 | Left | Somatomotor | LH_SomMot_3 | -37 | -21 | 16 | 0.024375 | 0.000975 |
| 20 | Left | Somatomotor | LH_SomMot_6 | -56 | -8 | 31 | 0.038603 | 0.002895 |
| 31 | Left | Dorsal Attention | LH_DorsAttn_Post_1 | -43 | -48 | -19 | 0.008183 | 0.000123 |
| 41 | Left | Dorsal Attention | LH_DorsAttn_FEF_1 | -31 | -4 | 53 | 0.027735 | 0.001525 |
| 58 | Left | Limbic | LH_Limbic_TempPole_2 | -45 | -20 | -30 | 0.024957 | 0.00123 |
| 83 | Left | Default | LH_Default_PFC_1 | -35 | 20 | -13 | 0.019713 | 0.000394 |
| 84 | Left | Default | LH_Default_PFC_2 | -6 | 36 | -10 | 0.001409 | 9.41E-06 |
| 86 | Left | Default | LH_Default_PFC_4 | -12 | 63 | -6 | 0.024012 | 0.00072 |
| 88 | Left | Default | LH_Default_PFC_6 | -6 | 44 | 7 | 0.024012 | 0.000682 |
| 122 | Right | Somatomotor | RH_SomMot_7 | 58 | -5 | 31 | 0.038603 | 0.002772 |
| 137 | Right | Dorsal Attention | RH_DorsAttn_Post_3 | 59 | -16 | 34 | 0.041354 | 0.003308 |
| 156 | Right | Salience/Ventral Attention | RH_SalVentAttn_Med_1 | 7 | 9 | 41 | 0.048789 | 0.004147 |
| 164 | Right | Limbic | RH_Limbic_TempPole_3 | 25 | -11 | -32 | 0.024375 | 0.000862 |
| 178 | Right | Control | RH_Cont_Cing_1 | 5 | -24 | 31 | 0.001409 | 1.41E-05 |

## **Figure S1.** Associations of age 38 and current subjective memory concern with cortical mean diffusivity. Uncorrected p-values are plotted for all 200 cortical parcels, with warmer color indicating lower p-values (i.e., more statistically significant). A. Association between age 38 subjective memory concern and cortical mean diffusivity is depicted. B. Association between current subjective memory concern and cortical mean diffusivity is depicted.


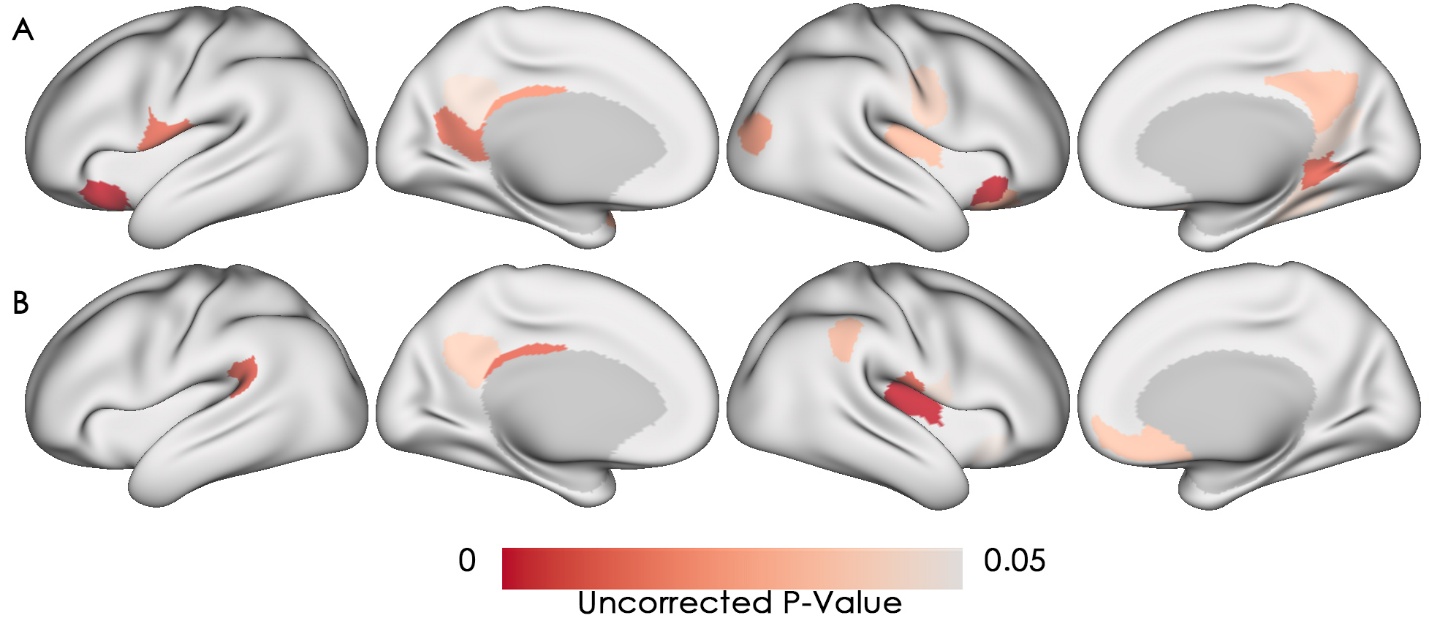


## **Figure S2.** Associations of objective memory with cortical mean diffusivity. Uncorrected p-values are plotted for all 200 cortical parcels, with warmer color indicating lower p-values (i.e., more statistically significant).


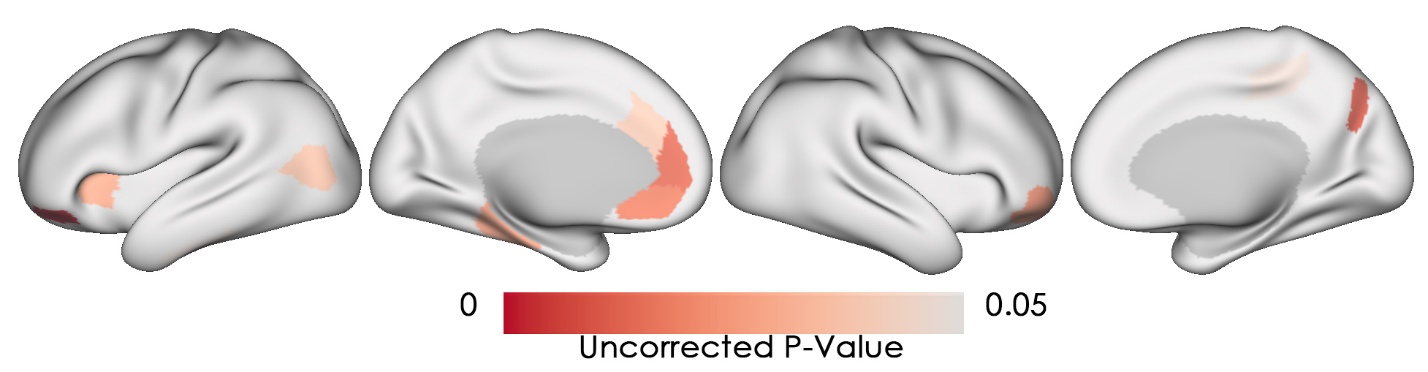


**Figure S3.** Associations of negative affect with cortical mean diffusivity. Uncorrected p-values are plotted for all 200 cortical parcels, with warmer color indicating lower p-values (i.e., more statistically significant). FDR-corrected p-values, A. Association between depressive symptoms and cortical mean diffusivity is depicted. B. Association between trait anxiety and cortical mean diffusivity is depicted.


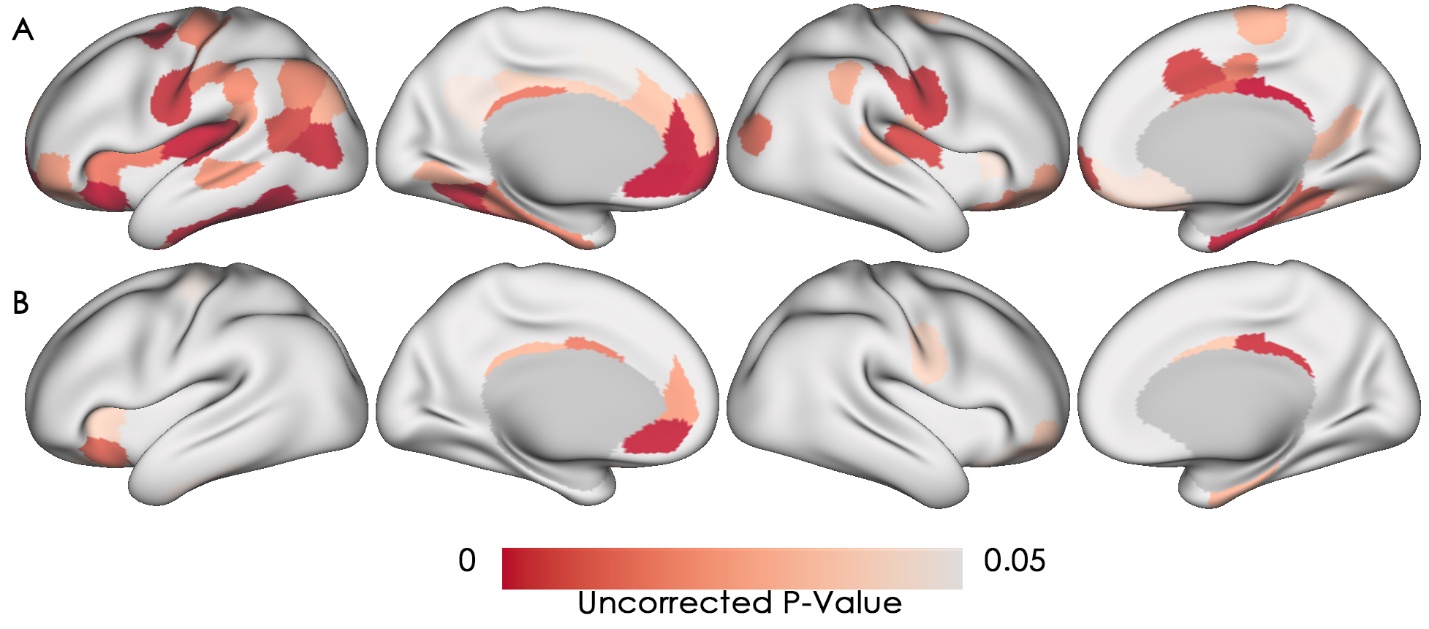


**Figure S4.** Associations of depressive symptoms with cortical mean diffusivity. FDR-corrected p-values are plotted for all 200 cortical parcels, with warmer color indicating lower p-values (i.e., more statistically significant).


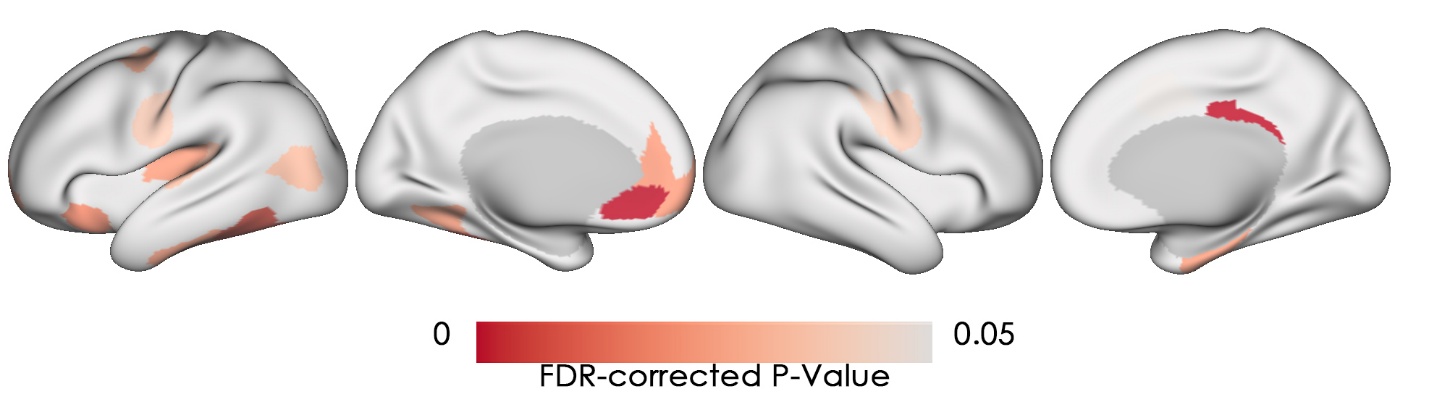

Supplement: Supplementary file 1 — (DOCX 529 KB) [file 11357_2025_1778_MOESM1_ESM.docx]
